# Virchow Testimonial Fund

**Published:** 1891-06

**Authors:** 


### Virchow Testimonial Fund.

On the 13th of October, 1891, when Professor Rudolph Virchow celebrates his 70th birthday, it is proposed to present him with a testimonial in recognition of his services to Medical Science. For the United Kingdom a strong Committee has been formed, with Sir James Paget as Chairman, Dr. Lauder Brunton as Treasurer, and Dr. Felix Semon and Mr. Victor Horsley as Secretaries.

Dr. Markham Skerritt, who represents this neighbourhood on the Committee, will be happy to afford any information on the subject, and to receive subscriptions.

